# Wrist-hand extension function recovery in spastic hemiplegia patient by botulinum toxin injection plus surface electromyography biofeedback therapy: A case report

**DOI:** 10.1097/MD.0000000000025252

**Published:** 2021-04-09

**Authors:** Zhang-xiang Wu, Chao Wang, Zheng Huang, Xue-han Liu, Mei Shen

**Affiliations:** Department of Rehabilitation Medicine, People's Hospital of Longhua, Shenzhen, Affiliated Hospital of Guangdong Medical University.

**Keywords:** biofeedback, botulinum toxin, case report, spasticity

## Abstract

**Rationale:**

Wrist-hand extension function rehabilitation is a vital and difficult part of hand function recovery in spastic stroke patients. Although botulinum toxin type A (BoNTA) injection plus post injection therapy was applied to the wrist-hand rehabilitation in previous reports, conclusion was inconsistent in promoting function. For this phenomenon, proper selection of patients for BoNTA injection and correct choice of post-injection intervention could be the crucial factors for the function recovery.

**Patient concerns:**

We reported a 46-year-old male suffered a spastic hemiplegia with wrist- hand extension deficit.

**Diagnoses:**

Computed tomography showed cerebral hemorrhage in the left basal ganglia region.

**Interventions:**

Four hundred units of BoNTA were injected into the spasticity flexors, and four-week post injection surface electromyography (sEMG) biofeedback therapy was applied to the patient.

**Outcomes:**

The patient exhibited post-intervention improvement in wrist-hand extensors performance (strength, range of motion, sEMG signals), the flexors spasticity, and upper extremity function.

**Lessons:**

The present case showed that 4-week of BoNTA injection plus sEMG biofeedback exercise improved the performance and function of wrist-hand extensors in the patient for short- and long-term. Proper selection of patients for BoNTA injection and correct choice of post injection exercise could play a vital role in the hand rehabilitation for patient with spastic hemiplegia.

## 1 Introduction

Wrist-hand extension function recovery is a vital and difficult component in hemiplegia rehabilitation. In patients with spasticity hemiplegia, wrist-hand flexors hypertonia is prevalent which would compress function of the extensors leading to limitations on activities of daily living (e.g., writing, drinking, picking, etc.).^[1–5]^ And most stroke patients would get permanent wrist-hand function disabilities if they could not get timely and effective healing for the critical window.^[6]^ Thus, an appropriate rehabilitation treatment program is believed to be extremely important for the patients.

To manage focal spasticity of muscles, botulinum neurotoxin type A (BoNTA) injection is commonly used in patients with spastic hemiplegia.^[7]^ BoNTA could produce transitory paralysis of the muscle through inhibition of local neuromuscular cholinergic transmission, which can relieve the disorders by spasticity. However, conclusion is inconsistent in improvement for hand function recovery through BoNTA injection plus post injection therapy in previous reports.^[8–11]^ For this phenomenon, different severity of spasticity in study-population and distinct post injection rehabilitation therapy in these researches could be the key factors. Thus, BoNTA injection for the proper patient and appropriate applied of post injection rehabilitation therapy may play a vital role in the result.

Based on this, we explored a treatment regimen of BoNTA injection plus proper post injection therapy for wrist-hand extension function recovery in proper patient with spastic hemiplegia. This case report is showing the beneficial effect of BoNTA injection plus surface electromyography (sEMG) biofeedback exercise in a mild stroke patient. In order to illustrate the research process simply, we mainly took the wrist extensors’ performance as the observing target for reflecting the extension function alteration at pre and postintervention.

## 2 Narrative

### 2.1 Case description

The patient was a 46-year-old male who suddenly felt right body dysfunction on November 14, 2019, previous with risk factors for stroke (e.g., diabetes, hypertension, chronic heart disease). Then, he was admitted to local hospital, blood pressure was recorded as 167/105 mm Hg. Computed tomography revealed cerebral hemorrhage with a 41×52×45 mm hyperechoic mass in his left basal ganglia region. And he worked as an engineer before the stroke. After participating inpatient routine rehabilitation for 8 weeks, he was able to ambulate solely without an assistive device. After discharge, he had received instruction of home rehabilitation exercise online at home during the COVID-19 epidemic control-period. On April 15, 2020, the patient was searching for medical help for his right wrist-hand dysfunction at our outpatient rehabilitation center. Meanwhile, routine inspection showed that he had a slight extension movement (grade 2-, manual muscle testing, MMT) in the right wrist and fingers, which were previously in flaccid paralysis. The patient reported that he almost exclusively to perform daily activities through his left upper extremity. He could not use his right hand to drink, write, eat, and dress. After case discussion by our multi-medical team, BoNTA injections combined with biofeedback training (E-link, Biometrics Ltd, UK) was recommended for the patient. And the patient responded to information about the cure arrangement. After the safety screening completed, written informed consent was signed by the patients to participate in this program.

### 2.2 Procedure

**Figure 1:**
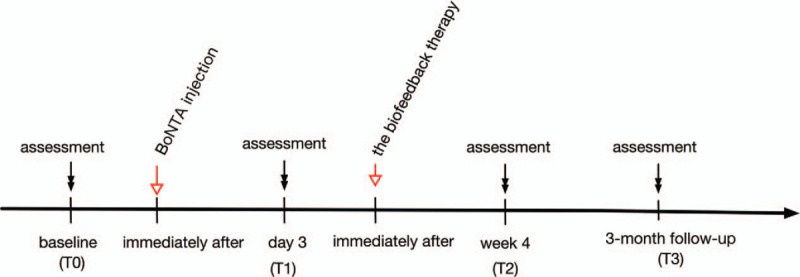
Time schedule of this study. BoNTA, botulinum neurotoxin type A.

Describe of time schedule of this research is shown in Figure 1. Before BoNTA injection, the patient received a baseline (T0) assessment in performance of the affect wrist-hand extensors, the spasticity, and upper limb function. Immediately after baseline assessment, the BoNTA injection was performed at our outpatient center (Fig. 1). Three days after the injection, the second (T1) assessment was fulfilled for observing the short-time treatment effect of BoNTA injection alone. And immediately after the second assessment, the sEMG biofeedback therapy was applied to which lasted for 4 weeks (Fig. 1). After that, the third (T2) and fourth (T3) assessments were respectively accomplished at week 4 and 3-month follow-up (Fig. 1).

### 2.3 Intervention

**Figure 2:**
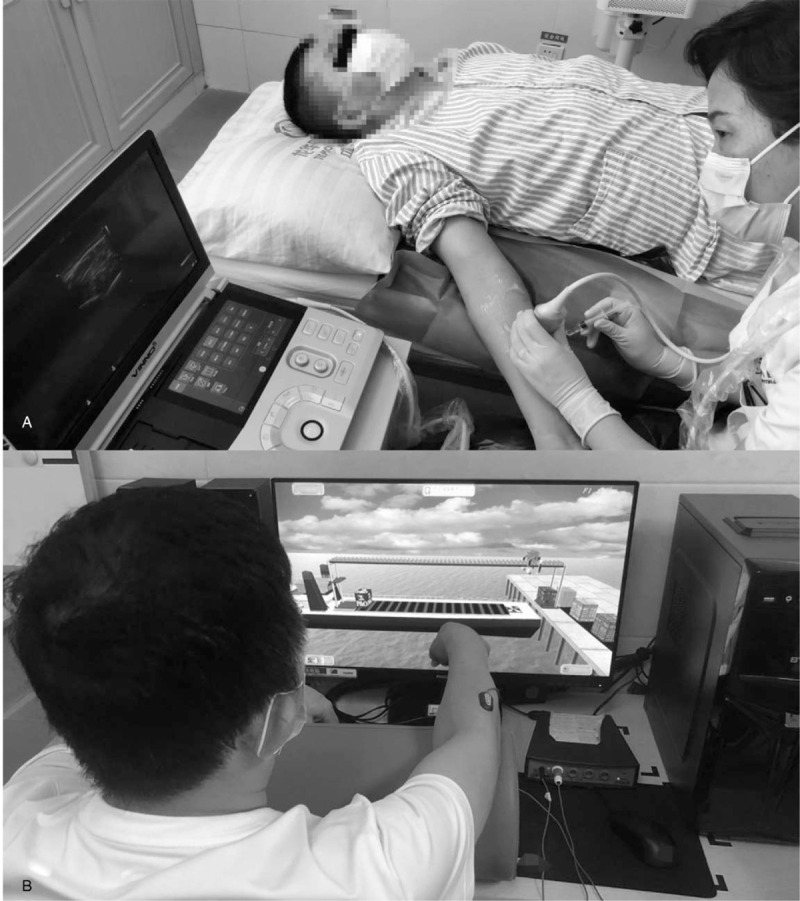
Botulinum toxin type A injection through ultrasound-guided (A), E-link biofeedback games training program (B).

The BoNTA injection was performed by an experienced physician (Shen Mei). The BoNTA (Hengli, China), supplied as a vacuum-dried powder in a 100-unit vial, was reconstituted with sterile normal saline (0.9%) to reach a total volume of 2.5 ml per vial. Muscles chosen for the injection were based on previous experience of BoNTA injection for spasticity.^[15]^ Four hundred units of BoNTA were injected into the belly of flexor carpi ulnaris, flexor carpi radialis, flexor digitorum profundus, flexor digitorum superficialis, flexor pollicis longus, pronator teres, palmaris longus, brachioradialis, biceps brachii, and brachialis at ten sites (Table 1). And the injections were accomplished through ultrasound-guided (Fig. 2a). After receiving 400 units of BoNTA injection in the wrist-hand flexors, a 4-week sEMG biofeedback computer game program was applied to the patient at our center for each day, 5 times weekly. While the biofeedback exercise intensity was set as 70% of the peak value when the extensors did maximum voluntary contraction. And 3 sets of games (each game for 7 minutes, game interval rest time for 1 minutes) was carried out for the patient (Fig. 2b). Furthermore, wrist-hand home exercise education was requested to the patient, which specific instructions of the home exercise was fulfilled by our therapists.

**Table 1:** Dose of BoNTA injection.

Injected muscle	Dose (unit)
flexor carpi ulnaris	50
flexor carpi radialis	50
flexor digitorum profundus	50
flexor digitorum superficialis	50
flexor pollicis longus	30
pronator teres	30
palmaris longus	30
brachioradialis	20
biceps brachii	80
brachialis	10

### 2.4 Assessment

Surface electromyography (sEMG) system (Biometrics Ltd, UK) was utilized to monitor the active activity of the wrist-hand extensors in this study. The patient performed a continuous wrist-hand extension of isometric maximum voluntary contraction for 5 seconds after receiving the instruction by the evaluator (Wang Chao). The assessing procedures were repeated 3 times with a time interval of 30 seconds prior to the next repetition. The electromyography signals from the muscles were collected through electrodes put on the skin surface in an air-conditioned room maintained at 22 to 25°C. Then, the electromyography signals were amplified ten times and bandpass filtered from 10 to 500 Hz at a sample frequency of 1000 Hz. After signals containing noise discarded, root mean square (RMS) value, integrate electromyograph (IEGM) value, and median frequency (MF) of the power spectrum were calculated. In addition, maximum strength of the wrist-hand extensors and active range of motion (a-ROM) of the extension were also measured in this study. For assessing the upper limb function change, the Wolf Motor Function Test (WMFT) was used in this research. The WMFT scale is a global functionality evaluation of the upper limb, which is developed for people with mild to moderate stroke.^[16]^ The scale contains 15 upper-extremity functional tasks to assess movement components required for daily tasks, each item score from 0 to 5. Two sections of WMFT (time scores and function scores) could be utilized to evaluate the patient's upper limb function. In this study, we only used the functional section of the WMFT. In addition, the Modified Ashworth Scale (MAS) was utilized for evaluating the wrist-hand flexor spasticity. And the Min-Mental State Examination (MMSE) was applied to perceive any cognitive changes which may affect training or test performance in this study.

### 2.5 Outcomes

**Figure 3:**
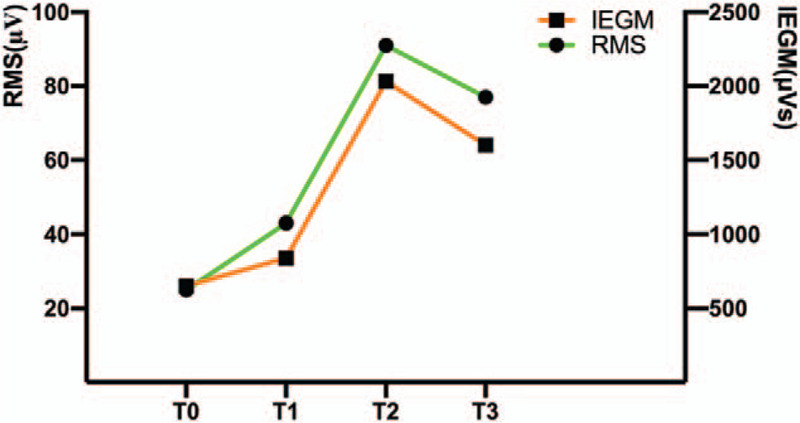
RMS and IEGM value variation in different assessing time. RMS = root mean square, IEGM = integrate electromyography, T0 = baseline, T1 = day 3, T2 = week 4, T3 = 3-month follow-up.

**Figure 4:**
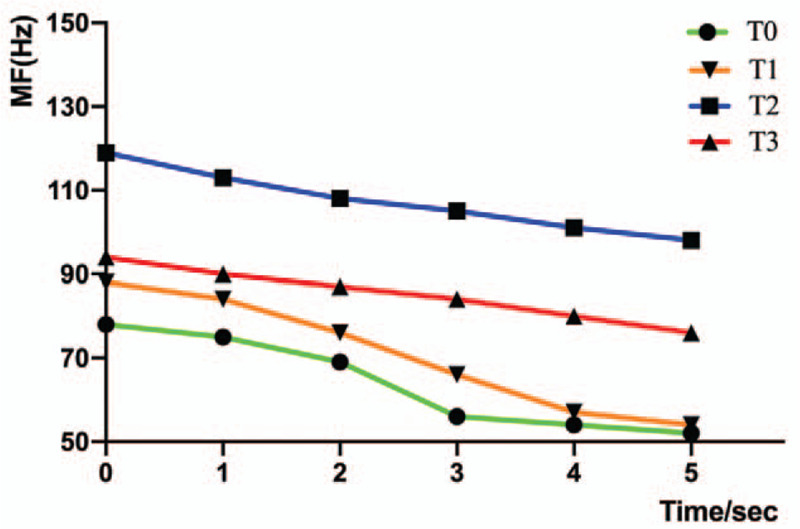
MF value variation during five-second isometric maximum voluntary contraction for different assessing time. MF = median frequency, T0 = baseline, T1 = day 3, T2 = week 4, T3 = 3-month follow-up.

Value alteration for RMS, IEMG, MF, WMFT, strength, a-ROM, and MAS at baseline (T0), day 3 (T1), week 4 (T2), and the 3-month follow-up (T3) are shown in Tables 2 and 3 and Figures 3 and 4. An apparent RMS value increase was found at T1 (43 μV), T2 (91 μV), and T3 (77 μV) compared with T0 (25 μV) (Table 2). Similarly, IEMG value was improved at T1 (841 μVs), T2 (2032 μVs), and T3 (1601 μVs) compared with T0 (652 μVs) (Table 2). And higher MF value was observed at T1 (71 Hz), T2 (107 Hz), and T3 (81 Hz) compared with T0 (64 Hz) (Table 2). Meanwhile, higher mean values and smoother downtrend were revealed after post treatment during 5 seconds isometric maximum voluntary contraction (Fig. 4). Regarding to WMFT score alteration, the patient initial mainly task performance deficit was on the last 8 items (all was 1 score) (Table 3). Although a non-noticeable item change was found at T1 (2.13 scores) comparison with T0 (2.07 scores), the overt alteration was exhibited at T2 (2.80 scores) and T3 (3.06 scores) (Table 3) which minimally clinically important difference of WMFT was reported to be 0.4 scores.^[11]^ Moreover, the a-ROM of the wrist-hand extension was also improved at T1 (−5°), T2 (27°), and T3 (25°) compared with T0 (−15°) (Table 2). And the strength of wrist-hand extension was exhibited for 10.6N at T2 and 8.6N at T3, although the patient cannot complete the measurement task at T1 and T0 (Table 2). The wrist-hand flexors spasticity was improved at T1 (grade 1+, MAS), T2 (grade 1, MAS), and T3 (grade 1+, MAS) compared with T0 (grade 2, MAS) (Table 2). Furthermore, the patient's MMSE score remained constant, which means that he had no cognitive changes that may interfere with the training or test performance throughout the study period.

**Table 2:** Outcome measures after the intervention.

	T0	T1	T2	T3
Active ROM of wrist extension (°)	−15	−5	27	25
Active wrist extensors strength (N)	/	/	10.6	8.6
MAS for wrist flexion (score)	2	1+	1	1+
sEMG of wrist extensors RMS (μV)	25	43	91	77
MF (Hz)	64	71	107	85
IEGM (μVs)	652	841	2032	1601

Data present as mean score. ROM = range of motion, sEMG = surface electromyography, RMS = root mean square, MAS = Modified Ashworth Scale, WMFT = Wolf Motor Function Test (functional section), IEGM = integrate electromyography, MF = median frequency, T1 = baseline, T1 = day 3, T2 = week 4, T3 = 3-month follow-up, a diagonal line (/) means measurement cannot be accomplished.

**Table 3:** Score alteration of Wolf Motor Function Test (WMFT).

Item	T0	T1	T2	T3
Forearm to table (side)	4	4	4	4
Forearm to box (side)	3	3	3	4
Extend elbow (side)	4	4	4	4
Extend elbow (weight)	3	3	3	3
Hand to table (front)	3	3	4	4
Hand to box (front)	3	3	3	4
Reach and retrieve	3	3	3	3
Lift can	1	2	3	4
Lift pencil	1	1	3	3
Lift paper clip	1	1	2	2
Stack checkers	1	1	2	2
Flip cards	1	1	2	2
Turn key in lock	1	1	2	2
Fold towel	1	1	2	3
Lift basket	1	1	2	2
Mean Score	2.07	2.13	2.80	3.06

Fifteen upper limb functional tasks are incorporated to assess movement components required for daily tasks. Quality of movement is assessed with scores ranging from 0 to 5. 0 = not attempted, 1 = attempt made, but not participating functionally, 2 = movement does participate, but needs more than 2 attempts, assistance, compensatory movements, or performing task very slowly, 3 = movement does participate, but affected by synergy or finishing task slowly, 4 = movement approximately normal, but slightly slower, or lacking fine coordination, 5 = normal movement, T0 = baseline, T1 = day 3, T2 = week 4, T3 = 3-month follow-up.

## 3 Discussion

This case report aimed to illustrate the effect of BoNTA injection plus sEMG biofeedback training on wrist-hand extension function recovery through observing wrist extensors performance and function improvement in patients with spastic hemiplegia. In this study, the value of strength, a-ROM, WMFT, MAS, and sEMG signals were improved after the combined intervention compared with baseline (Table 3). Thus, 4-week BoNTA injection plus sEMG biofeedback therapy had achieved improvement for the extensor's performance, upper extremity function (especially the section of hand function), and the spasticity in short-and long-term.

The results of studies on BoNTA injection alone or which combined with post injection therapy for motor function recovery are inconsistent in previous literature. Pennati et al reported robotic training combined with BoNTA injection had achieved noticeable improvement in relieving spasticity and enhancing upper limb function in stroke patients.^[17]^ Sun et al showed BoNTA injection plus modified constraint-induced movement therapy was also significantly effective in improving upper limb spasticity and function.^[18]^ However, it was also reported to limited evidence for improving active arm function, but improving basic upper extremity tasks (e.g., facilitation of dressing, hand hygiene) and pain.^[9–10]^ Meanwhile, Rosales et al reported the same results that BoNTA injection combined rehabilitation can provide a sustained reduction in early poststroke upper extremity spasticity, but arm and hand function were not affected.^[11]^ Shaw et al ^[10]^ argued that agonist active weakness may be the critical factor rather than restriction by the spasticity. Regarding the contradiction, multi-factor may be the cause. Different severity of spasticity in study-population and distinct postinjection rehabilitation exercise may be the key influencing factors in these reports. And the optimal BoNTA dose and injection position have not reached an agreement for the affected upper limb yet, although BoNTA injection had been commonly used for the management of spasticity in stroke patients.

From our results, an apparent sEMG and active ROM improvements were found only 3 days after the injection, although the biofeedback motor training was not applied yet. Thus, wrist-hand extensors recruitment was indeed restricted by the spastic flexors in the patient. Regarding to dose of BoNTA and selection of proper patient, 400 units of BoNTA was injected and mild spasticity patient were chosen in our research. Based on our long-term clinical experience, 2 important points need to be paid attention to: firstly, BoNTA injection in appropriate patients who are supposed to remain the weak agonist strength for grade 2- (MMT) at least; secondly, the dose of BoNTA should be sufficient which about 400 units were recommended for antagonist spasticity of grade 2-3 (MAS) and about 300 units for the grade 1^+^ - 2 (MAS) which supported by previous article.^[15]^ Furthermore, previous studies recommended wrist-hand extensor contraction facilitating should be conducted as early as possible which is important to promote the whole hand or upper limb function recovery.^[3,4,19]^ The view we hold is that exercises for the weak agonists are essential after injection of BoNTA. Otherwise, we may lose the best opportunity to achieve rehabilitation goals during “gold time” in motor function recovery.

Thus, a proper post injection rehabilitation training is vital. And evidence showed BoNTA injection plus post rehabilitation achieved a more remarkable improvement compared with BoNTA injection alone.^[8]^ In this study, we adopted sEMG biofeedback exercise as the post rehabilitation intervention. The sEMG biofeedback exercise is a flexible specific-task hand rehabilitation method which could train precisely on the target muscle through using sEMG for training and biofeedback. And it can be tailored for specific patient needs in isometric strengthening, motor learning, and control.^[12]^ In addition, previous studies exhibited that sEMG biofeedback exercise was more effective than conventional physiotherapy.^[13,14]^ For this phenomenon, a probable interpretation is that sEMG biofeedback can effectively promote the accuracy of training. Thus, the sEMG biofeedback training can improve the weak agonist performance more accurately in patients with stroke spasticity, which can better improve its function.

In summary, although BoNTA injection is widely used for the management of stroke spasticity in patients, the BoNTA injection combined with a sEMG biofeedback motor rehabilitation could make the patients’ benefits enlarge. Also, evaluation of proper patients for BoNTA injection should be paid attention to. Finally, a large-sample and randomized controlled trial research needs to be supplemented in the future to obtain more evidence for the conclusion.

## 4 Conclusion

This case report showed that 4-week of BoNTA injection plus sEMG biofeedback training significantly improved the performance and function of antagonist of the spastic-muscle for hand rehabilitation in patient with spastic hemiplegia in short- and long-term. Proper selection of patients for BoNTA injection and correct choice of post injection exercise could play a vital role in hand rehabilitation.

## Author contributions

**Conceptualization:** Mei Shen.

**Data curation:** Zhang-Xiang Wu, Chao Wang.

**Formal analysis:** Zhang-Xiang Wu, Chao Wang.

**Methodology:** Chao Wang, Zheng Huang, Xue-han Liu.

**Project administration:** Zhang-Xiang Wu, Chao Wang, Mei Shen.

**Resources:** Mei Shen.

**Supervision:** Mei Shen.

**Writing – original draft:** Zhang-Xiang Wu, Chao Wang, Zheng Huang, Xue-han Liu, Mei Shen.

**Writing – review & editing:** Zhang-Xiang Wu, Mei Shen.
